# The Medico-Legal and Social Aspects of the Eligibility Examination for Enrolment in the Seafarers Registry: A Single-Center Retrospective Study

**DOI:** 10.3390/healthcare12232410

**Published:** 2024-11-30

**Authors:** Antonietta Porzio, Alessandro Feola, Edoardo Mazzini, Angelo Lauro, Maria Rosaria Forte, Marco Trabucco Aurilio, Carlo Pietro Campobasso

**Affiliations:** 1 Department of Mental and Physical Health and Preventive Medicine, University of Campania “Luigi Vanvitelli”, Via Luciano Armanni 5, 80138 Naples, Italy; 2 Department of Occupational and Environmental Medicine, Epidemiology and Hygiene, National Institute for Workers’ Compensation (INAIL), 00143 Rome, Italy; 3 Department of Experimental Medicine, University of Campania “Luigi Vanvitelli”, Via Luciano Armanni 5, 80138 Naples, Italy; 4 Regional Directorate Campania, National Institute for Insurance Against Accidents at Work (INAIL), 80143 Napoli, Italy; 5 Medico-Legal General Coordination Office INPS, 00143 Rome, Italy

**Keywords:** seafarers, health knowledge, occupational risk, fitness to work, maritime medicine

## Abstract

Background: Working on board presents unique healthcare challenges for maritime personnel. Italian Law mandates a medical fitness assessment for seafarers registry enrolment. The Port Health Officer assesses seaworthiness and, against this judgment, the applicants can appeal to the First-degree Medical Commission. Studies on maritime personnel’s medical fitness are limited and primarily focus on the health conditions of individuals already employed in the maritime sector. Methods: A retrospective study reviewed 459 medical reports of 361 seafarers from Naples’ Port Authority First-degree Medical Commission (2013–2022). Characteristics such as sex, age, diseases, and suitability judgments were analyzed. Results: Out of the 361 candidates, most were male with an average age of 28.67 years. A total of 160 (44.32%) were deemed suitable for matriculation in both the first and second categories, 79 (21.88%) were approved for one category, 53 (14.68%) were found not suitable, and 69 (19.11%) are still under assessment. Eye diseases were most common (50.83%), followed by cardiovascular and orthopedic conditions. Applicants with poorly controlled diabetes mellitus, epilepsy, psychiatric disorders, and advanced tumors were declared unsuitable. Conclusion: Our study provides opportunities to improve applicants’ awareness of the physical requirements for pursuing a career in navigation and to update the list of illnesses and physical impairments used for this medico-legal evaluation.

## 1. Introduction

Maritime transport plays a leading role in the Italian economy. During the period of 2020–2021, Italy ranked second in the European Union regarding the volume of gross maritime cargo handled [1] and remained the leading country, alongside Greece, for seaborne passenger transport. “The EU blue economy report 2021” of the European Commission revealed that Italy’s maritime economy sector employed approximately 530,000 people, with a substantial increase in employment in Maritime Transport (+52.3%), compared to other European countries [2]. This reflects the significant reliance on maritime activities as a key driver of the country’s economic growth.

Working on board presents unique healthcare challenges for maritime personnel. They may experience special health issues and emergencies due to limited access to immediate healthcare, confined and partially enclosed spaces, and specific working conditions [3].

Maritime employment is also characterized by a high incidence of accidents [4]. According to the Italian National Institute for Insurance against Workplace Accidents (INAIL) dataset [5], between 2017 and 2021, an annual average of approximately one thousand work-related accidents were claimed to the INAIL by maritime personnel, of which approximately 70% were recognized. In the INAIL Second Report on maritime workers [6], a total of 9298 accident cases involving maritime workers between 2004 and 2022 was reviewed. The highest number of accidents occurred among deck personnel, followed by non-commissioned officers, hotel/service crew, and engine room staff. Most accidents took place during deck operations (22%), engine room tasks (16%), or cabin/kitchen duties (17%). The most common causes of accidents were falls and incidents related to mooring equipment, ropes, winches, and ladders. The COVID-19 pandemic emerged as a significant new cause from 2020 onward. People aged 50 and over are at a higher risk of accidents compared to other age groups. Workers under 30 years old accounted for 24% of accidents on ferries, 14% on fishing vessels, 21% on cruise ships, and 30% on other types of vessels. From 2017 to 2021, most occupational diseases reported came from the maritime fishing sector, with an annual average of 853 cases. Most of these diseases were related to musculoskeletal disorders. Pleural and peritoneal mesothelioma and ear disorders, including deafness, are the most prevalent in the maritime and inland waterway transport sector, followed by musculoskeletal disease, asbestosis, and respiratory illness [5].

According to the Centre for Maritime Safety and Health Studies, mortality rates are significantly elevated onboard vessels compared to occupations on shore [7]. Therefore, preventing injuries and occupational diseases among seafarers is crucial for their safety and well-being. The occurrence of accidents at work may be predicted by assessing an individual’s physical fitness, hypothesizing that employees in good health are less prone to experiencing accidental events [8]. Seafarers must be physically and mentally prepared to handle the unique challenges of maritime activities and to manage stress [9] and emergency situations. They should maintain high alertness and performance levels, thereby reducing risks related to fatigue, distraction [10], or unmanaged medical conditions. Regular health surveillance at enrolment and at work, and a safe work environment could significantly lower the number of accidents at work and occupational diseases.

Therefore, evaluating the pre-boarding health status of the applicant is crucial for preventing potential diseases associated with maritime environments. In Italy, aspiring seafarers must undergo mandatory medical examinations for enrolment in the seafarers registry [11,12]. Maritime personnel, after the submission of an application form, undergo a medical examination conducted by the Seafarer Doctor as representative of the Maritime, Air and Border Health Office (USMAF). The goal of this examination is to ascertain that the applicant fulfills the psycho-physical requirements provided by the Royal Decree Law No. 1773 of 1933. If the Seafarer Doctor declares the applicant seafarer unsuitable, the candidate can appeal to the First-degree Medical Commission (FMC) that reviews medical records and, after a clinical examination, assesses a final judgment of suitability, unsuitability, or partial suitability [12].

Research on maritime personnel’s medical fitness is limited, primarily focusing on individuals who have already passed pre-boarding medical examinations and subsequently manifest or develop a medical issue during their shipboard experience [13,14,15]. Currently, there are no studies in the scientific literature that analyze the physical and psychological fitness required to start a maritime career. So, the aim of this study is to analyze these biological requirements for pursuing a career in navigation, enhancing candidates’ awareness for their future job prospects.

A sample study of 361 seafarer candidates who underwent the FMC’s medical examination was analyzed, focusing on the health conditions that might compromise medical eligibility for maritime employment following the national regulations for the medical examination of seaworthiness [11,12]. So, before moving to the other sections of this paper, a brief look at the main Italian legislative references is necessary. These rules report the categories of maritime activities and the physical impairments or health conditions that disqualify applicant seafarers from enrolment in each category.

### 1.1. The Italian Code of Navigation

The Italian Code of Navigation was firstly approved by the Royal Decree of 30 March 1942, No. 327 and recently updated to the Legislative Decree of 22 April 2020, No. 37 [11]. The provisions about the organization and the matriculation of seafaring personnel are reported in Title IV of the first part of the Code of Navigation. According to Articles 114 and 115 of the Code of Navigation, maritime personnel include seafarers, port service staff, and technical personnel of shipbuilding. Seafarers are divided into three categories:

1. Officers and low force personnel assigned to deck services of the engine and generally to technical services on board (first category);
2. Personnel assigned to supplementary services on board (second category);
3. Personnel assigned to local traffic and coastal fishing (third category).

Based on Article 119 of the Code of Navigation, Italian citizens aged 16 to 25, meeting the category-specific requirements, may register in the seafarer records. However, some exceptions are allowed for European citizens and for individuals exceeding the specified age limits. The removal of individuals from the seafarer records can occur in the instance of the death of the worker or his will to abandon the maritime activity, the loss of Italian citizenship, a permanent loss of physical requirements for navigation, or a conviction with a relative judgement for any crimes that prevent registration in the rolls.

Seafarers are eligible to receive a seaman’s discharge book (art. 122): this document contains personal information and details about any qualifications, honors, blood type, completed courses, and navigation experience on different types of merchant ships. It is considered a valid identity document and passport for all maritime-related activities and allows a seafarer to legally practice their profession (art. 220 of the Presidential Decree No. 328 of 15 February 1952—Regulations for the Implementation of the Navigation Code).

Additional physical and professional requirements for each maritime category are reported by the Decree of the Minister of Infrastructure and Transport 25 July 2016 [16] and Annex A of Presidential Decree no. 231 of 18 April 2006 [17]. They are summarized in Table S1 of the Supplementary Materials.

### 1.2. The Royal Law Decree of 14 December 1933, No. 1773

The regulations regarding medical examination of seaworthiness are contained in the Royal Law Decree of 14 December 1933, No. 1773 (“Assessment of the physical fitness of first-category seafarers” as modified by Law 28 October 1962, n. 1602 and Presidential Decree 30 April 2010, n. 114) [12]. According to this law, a seafarer undergoes a control visit by the Office of Maritime Health for the issue of certificates of seafarer eligibility examination for enrolment in the matriculation; a biennial check-up; a prior boarding visit; and a check-up after disembarkation due to illness and other medical examination requested by the shipowner.

When applying for registration, all categories of seafarers must undergo an evaluation by the Seafarer Doctor. He is authorized to assess and certify the suitability for the enrolment in the seafarer records. The medical examination can be made after completing a form at the USMAF Territorial Unit clinic. The form collects the personal data of the applicant seafarers and a medical history questionnaire, filled out in the presence of a physician. Blood and instrumental analyses are also required. The USMAF is in charge of providing the verification of physical and mental suitability for individuals intending to enroll in the seafarer registry and for port workers, and providing medical visits to assess physical suitability to engage in underwater fishing operations and diving activities.

The health conditions and physical impairments that exclude seafarer’s eligibility for enrolment in the first category registry, as listed in Royal Law Decree 1773/33, are reported below in Table S2 of the Supplementary Materials.

The Royal Law Decree’s List has not been changed since 1933, with the exception of conditions pertaining to the visual system. For visual impairment, the new requirements are reported in Rule A-1/9 of the STCW [18] (reported in Table S3 of the Supplementary Materials) as referred by the Legislative Decree no. 71/2015 [19].

If the Seafarer Doctor expresses a negative evaluation of the seafarer’s suitability, they can appeal to the FMC. The Commission’s members include three physicians: the Seafarer Doctor as President, a representative of the National Institute for Social Security (INPS), and the other one as an INAIL representative. Based on the medical records and the results of the clinical examination, the Commission assesses a final medico-legal judgment of suitability, unsuitability, or partial suitability. Against the FMC’s judgment, a seafarer can appeal to the Second-degree Central Medical Commission within sixty days. This assessment is the final one and no appeal is allowed.

### 1.3. The Law No. 113 of 23 September 2013

With Law No. 113 of 23 September 2013 [20], Italy formally ratified the Maritime Labour Convention (MLC, 2006), adopted by the International Labour Organization (ILO) on 23 February 2006 [21]. In this way, Italy aligns its national regulations with the international standards established by the MLC, which aims to safeguard the rights and welfare of maritime workers. The MLC Title 1 (“Minimum conditions required for seafarers’ work on board a ship”) stated that the competent authority must verify a seafarer’s fitness through valid medical certificates before they start working on a ship. The authority determines the examination and certificate requirements, considering international guidelines, to ensure accuracy.

## 2. Materials and Method

A retrospective analysis of seafarer medical examinations performed at the FMC of the Port Authority of Naples was conducted. The Port of Naples is one of the largest seaports in Italy with an annual traffic capacity of around 25 million tons of cargo, and it serves as a tourist hub for 10 million people annually. Furthermore, in Campania, there is the highest number of maritime institutions in Italy, with over 40 nautical secondary schools.

All the reports related to the Commission’s activities from 2013 to 2022 were reviewed. The timeframe was selected to encompass a period of approximately one decade, providing a comprehensive overview of the seafarer examination process over a significant period. A total of 459 medical reports on seafarer candidate examinations were available for analysis. These reports were systematically reviewed by two authors. No filter was applied during the selection phase and all reports were included. Through this process, a comprehensive dataset was established: for each case, the primary variables of interest were gender, age, diagnosed diseases, and the final judgment on the suitability assessment. The disease variable was encoded utilizing distinct numerical values corresponding to the various body systems affected. No additional information was extracted from the reports. The medical reports reviewed dealt with 361 seafarer candidates in total. A number of 84 of them were examined two or three times by the FMC.

Seafarers’ medical records were anonymized and protected from unauthorized access. Although the individuals were unaware of the review of their medical records in research studies like this one, the information was not subjected to illegal manipulation, which is contrary to research ethics and the international code of ethics for occupational health professionals.

## 3. Results

From January 2012 to December 2022 a total of 361 aspiring seafarers were examined by the FMC. All aspiring seafarers were Italian, except for one coming from Eastern Europe. Most of the individuals were males. Only 36 females were accounted for, highlighting a considerable gender disparity within the maritime sector. The mean age of the study sample was 28.67 years; the youngest aspiring seafarer was 16 years old, while the oldest one was 62 years old. All cases were categorized into four groups based on the FMC’s judgment as shown in Table 1: (1) suitable for matriculation for both the first and the second category; (2) suitable for matriculation for the first or the second category; (3) not suitable for matriculation; and (4) currently under assessment.

A total of 160 out of the 361 aspiring seafarers (44.32%) met the admission criteria for both the primary and secondary categories. A total of 79 individuals (21.88%) were assessed as suitable for only one of these categories and 53 applicants (14.68%) failed to meet the requirements for either category. The suitability of 69 candidates (19.11%) remains undetermined, as they are still undergoing evaluation. For these cases, additional medical records (i.e., further laboratory analyses, test results, or specialist evaluations) have been requested to provide a more comprehensive assessment of the individual’s health status.

Most of the 325 males (149 in total) were suitable for matriculation for both the first and the second category; 45 males were found to be unfit for matriculation as seafarers. Approximately half of the female participants were considered suitable, while eight women had restrictions for one of the two categories, and eight were unsuitable for matriculation. The mean age of suitable individuals was 26.74. The mean age was higher, up to 34.94 years, for unsuitable candidates.

The most reported diseases deal with the eye system. A total of 183 of seafarer applicants (50.83%) were affected by a vision disorder: 149 individuals (41.39%) showed refractive defects and 29 (8.6%) had dyschromatopsia. Most seafarers with dyschromatopsia were assessed as suitable only for the second category. Only five applicants were affected by other eye diseases like corneal leucoma, pseudophakia, microphthalmia, esotropia, or keratoconus: in one case, only an unfitness judgment was declared.

Cardiovascular conditions conflicting with seafarer enrolment were found in five cases of unsuitability (see art. 29 in Table S2 of the Supplementary Materials). Among these, three individuals were young females: two of them showed heart valve diseases and the other one had an atrial septal aneurysm without interatrial shunting.

Orthopedic diseases are reported in art. 12, 13, and 14 of the illnesses and physical impairments list (Table S2 of the Supplementary Materials). Among them, muscle hernias, ruptures, contractions, or aponeurosis adhesions that limit movement are included along with chronic changes of bones, joints, and surrounding tissues that result in significant congenital or acquired deformities and mutilations. Thirty candidates showed an orthopedic disorder but unsuitability to seafaring was assessed by the FMC in only eight cases due to bone fractures, spinal disorders, ankylosis, or finger amputation.

Four applicants were found to be suffering from diabetes mellitus that is listed as an unsuitability condition (see art. 4 of the Royal Law Decree—Table S2 of the Supplementary Materials). However, two applicants were considered fit for matriculation since they were under treatment with oral hypoglycemic drugs, showing good metabolic control. The remaining two cases, aged 22 and 60 years old, were unsuitable as they suffered from poorly controlled hyperglycemia with vascular complications.

Obesity is also a metabolic disorder reported in the list of the Royal Law Decree as a condition for unsuitability (art. 3—Table S2 of the Supplementary Materials). In the sample study, a 22-year-old male, underwent three visits to the FMC for obesity. At the first visit, a body weight of 197 kg (BMI 54.57) was found, and a judgment of unsuitability was declared. Following a sleeve gastrectomy procedure, their BMI was reduced to 32.1, but he was still deemed temporarily unfit. In the last evaluation, the candidate was found to be suitable for the first category only.

A single case of epilepsy was reported in the sample study. The young male was judged unsuitable according to art. 18 (Table S2 of the Supplementary Materials) as well as another applicant suffering from a psychotic syndrome and hypomania (art. 17—Table S2 of the Supplementary Materials).

Hearing impairments suitable for seafaring were found in two individuals since they are unsuitable (art. 24—Table S2 of the Supplementary Materials) only if a bilateral hearing loss occurs and when the voice is not perceived at less than 5 m with a whisper or in case of unilateral hearing loss when the voice is not perceived at less than 1 m.

Eight out of 361 individuals suffered from a neoplastic disease. Three of these were considered eligible because of the low grading and staging of the tumors surgically removed (thymic hyperplasia, pulmonary hamartoma, and follicular adenoma). The other three cases were unsuitable according to art. 11 (malignant tumors and benign tumors that, due to size, number, or location, cause noticeable deformity or impede movement freedom and the function of an important organ, thus markedly reducing work performance). These individuals were affected by malignant neoplasms in the advanced stage or with secondary complications that precluded extended periods at sea. The three cases regard an Ewing’s sarcoma of the ninth rib undergoing radiation treatment, with a recurrence in 2016 managed through chemotherapy; a second-grade astrocytoma treated with antiepileptic drug and a follicular variant papillary carcinoma that had been addressed through complete thyroidectomy and currently under hormonal therapy.

## 4. Discussion

Worldwide, there has been limited research on medical fitness among maritime sector personnel. Most of them are dealing with employers already recruited to work at sea that already passed the pre-boarding clinical examination. A Dutch study, examining 7617 seafarer medical evaluations, revealed a high rate of temporary unfitness due to cardiovascular issues and vision disorders with a low rate of permanent unsuitability (0.6%). [13]. A retrospective cross-sectional study in Benin [14] investigated the health profiles of seafarers undergoing medical examinations (pre-employment, periodical, or return to work) to identify conditions potentially impacting their medical suitability. More than 60% of seafarers were above 40 years old and suffered from hypertension and obesity, resulting in a high unrestricted fitness rate [14]. A 12-year observational study reported a very low incidence rate of permanent medical unfitness (<1%) among French seafarers from 2005 to 2016 [15]. Among the unfit seafarers, the major causes of unfitness included spinal rheumatological conditions, upper limb injuries, mental and behavioral disorders, and circulatory diseases [15]. Additional disorders can also affect seafarers such as chronic visual defects, hearing impairment, occupational deafness, venous insufficiency, varicose veins, and hemorrhoids. They can be related to inadequate preventive measures in the work environment but also to sedentary lifestyles and the lack of regular physical activity on vessels. [14] Such factors could be considered grounds for deeming an individual unfit for seafaring duties and life aboard ships.

Therefore, evaluating the pre-boarding health status of the applicant is crucial for preventing potential diseases associated with maritime environments. Several countries, including France, the UK, Poland, the Netherlands, and the Scandinavian countries require professional civilian seafarers to undergo a medical fitness assessment before employment [14]. Like in these countries, three types of medical examinations are mandatory: pre-employment, a return to work after a temporary disease, and periodic medical examination for health surveillance. In Italy, seafarer candidates must undergo mandatory medical examinations by the USMAF’s Seafarer Doctor. However, if the Seafarer Doctor declares the applicant seafarer unsuitable, the candidate can appeal to the FMC that is in charge to assess a final judgment of suitability, unsuitability, or partial suitability.

The results of our retrospective study on 459 medical reports of 361 seafarer candidates show a high rate of unsuitability to work at sea (14.68%). A total of 53 out of 361 candidates were assessed as unsuitable for all the three categories reported by the Italian Code of Navigation.

Among the 53 aspiring seafarers deemed medically unfit, 45 were male and 8 were female, with a total average age of 34.94 years. Most of these individuals (23%) presented with two or more pathological conditions contributing to the unfitness assessment. In 15% of cases, the unfitness was attributed to a severe refractive error that impaired the visual acuity of the aspiring seafarer. Two cases of dyschromatopsia were also reported. Cardiovascular conditions led to unsuitability in seven cases, involving three young females. A total of five cases of hematopoietic disorders were also documented: monoclonal gammopathy, congenital erythrocytosis under phlebotomy therapy due to VHL mutation, favism, iron-deficiency microcytic anemia under iron therapy, and bone marrow dysplasia. A total of 8 out of 30 applicants with orthopedic diseases and two applicants with poorly controlled hyperglycemia and vascular complications were deemed unfit. One candidate was disqualified for epilepsy; another was diagnosed with psychotic syndrome and hypomania and was thus disqualified. Out of the eight candidates with neoplastic conditions, three were not eligible due to non-surgically removed tumors with high grading and staging. For the 53 applicants deemed unfit by the aforementioned Commission, enrolment in the seafarer registry is prohibited unless they submit an appeal to the Second-Degree Central Medical Commission. For these candidates, if the judgment of unfitness is confirmed, the possibility of pursuing a future career as a seafarer would be precluded, forcing the individual to seek alternative employment opportunities and face the failure of their professional aspirations. This circumstance clearly entails substantial social and economic consequences.

The limitations of the study have been acknowledged: the selection of a descriptive observational study design necessitates the consideration of potential selection bias, and the results are derived from a restricted population within a specific timeframe and setting, which may not be generalizable to broader populations. Moreover, the paucity of similar scientific investigations in the extant literature precludes a comparative analysis of our findings with those obtained by other research groups. Nevertheless, the significant strength of the study is its exploration of a novel topic in the available scientific literature, thereby establishing a foundation for future research with the intention of updating our dataset with other regional context data in the near future.

It is worth mentioning that seafarers require more advanced knowledge and awareness including basic health care and emergency response skills, and they need targeted education and training tailored to the unique conditions onboard. There have been several studies on seafarer health knowledge and awareness. Grappasoni et al. [22] evaluated seafarers’ awareness of health risks through an anonymous questionnaire given to the crew members of nine tankers and the office staff of an Italian company operating in the maritime transport sector. This study reports that seafarers have a good knowledge of blood-borne and sexually transmitted diseases and express greater concern about psychological issues from isolation and the risk of inadequate care for disease or injury on board. In 2018, a Turkish study [23] enrolled 266 undergraduate students at maritime faculties to answer an anonymous and voluntary questionnaire with the aim of assessing different levels of health knowledge and seafarer risk awareness. The results of the study indicate that most maritime students possess adequate basic health knowledge but lack awareness and understanding of transmission risks, communicable diseases, and food hygiene, which is often limited to unreliable sources such as the Internet. A multicenter study on risk perception was conducted involving nine Italian port areas, and administered a questionnaire comprising 54 items: among the fishing sector’s workers, only 37.7% of respondents reported knowledge of the most prevalent occupational diseases, and 34.4% were aware of the most frequent causes of accidents [24].

However, there are no studies in the available literature that have explored the awareness of seafarer candidates regarding the diseases and other health conditions that could preclude them from starting a career in the maritime sector. Further research could examine the extent of awareness among applicants regarding medical conditions that render seafaring activities and maritime life incompatible.

Understanding the impact of maritime employment on health is essential for ensuring safety at work. A limited number of studies are also available regarding the assessment of health requirements for performing job activities in specific work contexts and environments, such as those of airplane pilots [25] and train [26] or truck drivers [27]. Extant studies have indicated that the conditions most frequently causing unfitness among train drivers are cardiovascular diseases, particularly arterial hypertension, as well as diabetes and obstructive sleep apnea [28]. Metabolic and cardiovascular diseases have been identified as potential factors that reduce future workers’ fitness to work as truck drivers [27]. However, due to the limited number of studies conducted so far, it remains challenging to provide a comprehensive understanding of the fitness conditions for specific worker groups, including seafarers. Therefore, it is evident that there is a need to increase research efforts on this topic to enhance the understanding of occupational health and to facilitate the implementation of health surveillance programs.

Recognizing the unique physical and psychological challenges of these types of work, particularly seafaring, enables workers to implement preventive strategies, such as regular medical examinations, adherence to safety protocols, and lifestyle modifications that promote health. This proactive approach safeguards maritime employees’ well-being and guarantees a safer and more productive work environment, benefiting both the workforce and the maritime industry.

## 5. Conclusions

The results of this study offer a representative point of view dealing with the eligibility medical examinations of seafarer candidates for work at sea made in one of the main Italian seaports. Based on our findings, the main factors that could potentially impair seafarers’ medical suitability for their work at sea are refractive defects, orthopedic and cardiovascular diseases, and neurological disorders. All these defects could lead to a judgment of unfitness, preventing seafarer applicants from starting a career in the maritime sector. Most of the seafarer candidates are minors or young people and if they are not physically or mentally suitable, it not only undermines their aspirations but also leads to a substantial waste of resources and financial investments. The efforts spent by families and the government to promote education and provide technical training may be in vain. A comprehensive approach to the training of future maritime professionals could be achieved through a structured informative network involving not only educational institutions but also families and health professionals. An overview of the physical requirements for the recruitment of seafarers pursuing a career in the navigation sector can be provided to maritime applicants. This can be made through periodical meetings in schools and communities or with specific school curricula to reach a larger number of students. Furthermore, promoting scientific research on this topic, potentially comparing these data with national and international experiences, could be valuable in offering a more comprehensive understanding of this particular issue.

Although nearly a century has passed since the list of illnesses and physical impairments provided by the Royal Law Decree 1773/1933, the physical criteria have remained largely unchanged. In the contemporary context, the application of such outdated criteria can be considered anachronistic, and they challenge the medico-legal assessment of fitness for navigation. New treatments and medical approaches to diseases and impairments can improve the occupational capacity of candidates that can be suitable for working at sea. Therefore, updating these lists will be necessary to ensure more equitable access to employment opportunities for candidates and, most critically, enhanced workplace safety.

## Figures and Tables

**Table 1:** Classification of all aspiring seafarers by First-degree Medical Commission’s judgment.

		Suitable for Matriculation for the First AND the Second Category (*n* = 160)	Suitable for Matriculation for the First OR the Second Category (*n* = 79)	Not Suitable for Matriculation (*n* = 53)	Currently Under Assessment (*n* = 69)
Gender					
	Male	146 (91%) *	71 (90%)	45 (85%)	63 (91%)
	Female	14 (9%)	8 (10%)	8 (15%)	6 (9%)
Age, years, mean (SD)					
	All cases	26.74 (11.35)	25.72 (9.35)	34.94 (14.25)	31.71 (12.71)
	Male	26.58 (11.07)	25.90 (9.60)	35.80 (14.56)	31.52 (12.74)
	Female	28.43 (14.31)	24.13 (6.94)	30.31 (12.01)	33.67 (13.38)
Eye disease					
	Refractive errors	69 (43%)	51 (65%)	8 (15%)	21 (30%)
	Dyschromatopsia	8 (5%)	13 (16%)	2 (4%)	6 (9%)
	Other eye disease	2 (1%)	1 (1%)	1 (2%)	1 (1%)
Cardiovascular disease					
	Hypertension	1 (1%)	-	-	2 (3%)
	Arrhythmia	-	-	1 (2%)	-
	Myocardial infarction	-	-	2 (4%)	1 (1%)
	Other cardiovascular disease	9 (6%)	1 (1%)	4 (7%)	5 (7%)
Respiratory disease					
	Asthma	1 (1%)	-	-	1 (1%)
	Other respiratory disease	2 (1%)	-	-	4 (6%)
Neurological and psychiatric disease					
	Epilepsy	-	-	1 (2%)	-
	Psychiatric pathology	-	-	1 (2%)	
	Other neurological disease	2 (1%)	-	-	1 (1%)
Gastrointestinal disease					
	Hepatic Cirrhosis	-	-	-	-
	Hepatic Steatosis	3 (2%)	1 (1%)	-	-
	Other gastrointestinal disease	-	1 (1%)	-	3 (4%)
Orthopaedic disease					
	Bone fractures	3 (2%)	-	1 (2%)	3 (4%)
	Spinal disorders	3 (2%)	-	3 (6%)	1 (1%)
	Other orthopedic disease	11 (7%)	-	4 (8%)	1 (1%)
Otolaryngology disease					
	Hypoacusia	2 (1%)	-	-	1 (1%)
	Other otolaryngology disease	1 (1%)	-	1 (2%)	-
Other disease					
	Endocrinopathies	5 (3%)	-	1 (2%)	-
	Obesity	-	1 (1%)	-	-
	Diabetes mellitus	2 (1%)	-	2 (4%)	-
	Urinary pathologies	2 (1%)	-	1 (2%)	-
	Rheumatological disease	1 (1%)	-	-	-
	Blood Hematopoietic disease	3 (2%)	-	5 (9%)	2 (3%)
	Neoplasm	3 (2%)	-	3 (6%)	2 (3%)
Two or more diseases **		24 (15%)	9 (11%)	12 (23%)	9 (13%)
Not available		3 (2%)	1 (1%)	-	5 (7%)

* Every percentage refers the total individuals of the single group reported in the vertical bars. The percentages containing a decimal fraction are rounded off to the next lower or higher whole number according to whether the decimal fraction is greater or less than 0.5. ** Where two or more conditions contributed equally to medical unfitness, the case is recorded in the horizontal bar designated as “two or more diseases”. However, if a single condition was determined to be the primary cause of unfitness, only that principal reason is retained.

## Data Availability

Data are contained within the article and Supplementary Materials.

## Supplementary Materials

The following supporting information can be downloaded at: https://www.mdpi.com/article/10.3390/healthcare12232410/s1, Table S1: Requirements of professional qualifications of maritime personnel; Table S2: List of illnesses and physical impairments that disqualify individuals from enrolment in the first category of the seafarers’ register; Table S3: STCW Code Table A-I/9 regarding the minimum in-service eyesight standards for seafarers.
