# Supplementary material for: The Medico-Legal and Social Aspects of the Eligibility Examination for Enrolment in the Seafarers Registry: A Single-Center Retrospective Study
Source: Healthcare (Basel). 2024 Nov 30;12(23):2410. doi: 10.3390/healthcare12232410 (PMC11641269; doi:10.3390/healthcare12232410)
Supplement: Supplementary file 1 [file healthcare-12-02410-s001.zip › Supplementary Material Table S1.pdf]

Supplementary materials:

Table S1. Supplemental materials: Requirements of professional qualifications of maritime personnel

**FIRST CATEGORY**	
**DECK PERSONNEL**	
Deck Boy	16 years old
Deck Hand	at least 12 months of sea service as a Deck Boy
Sailor	18 years old; at least 24 months of sea service (at least 12 months on deck)
Tanker Man for Oil Tankers	a specific training course for oil tankers and at least 24 months of sea service on oil tankers, either on deck or in the engine room as a Sailor or Ordinary Seaman; or have completed at least 12 months of sea service as a trainee tanker man
Tanker Man for Chemical Tanker	a specific training course for chemical tankers and at least 24 months of sea service on chemical tankers, either on deck or in the engine room as a Sailor or Ordinary Seaman; or have completed at least 12 months of sea service as a trainee tanker man
Tanker Man for Gas Carriers	a specific training course for gas carriers and at least 24 months of sea service on gas carriers, either on deck or in the engine room as a Sailor or Ordinary Seaman; or have completed at least 12 months of sea service as a trainee tanker man
Deck Laborer	at least 12 months of sea service with a qualification as a mechanical worker, carpenter, or motorist laborer
Tractor Driver	a D/E driver's license and provide suitable documentation from a previous employer proving at least 24 months of work involving tasks requiring the aforementioned license
Cabinet maker	at least 3 years in furniture construction companies; or have completed 24 months of sea service on deck, including at least 12 months assisting the cabinet maker
Carpenter	performed specific work for at least 3 years in shipbuilding, repair, or outfitting establishments; or hold the qualification of ship's carpenter; or have completed at least 3 years of sea service on deck and in the engine room; or have completed at least 24 months of sea service as a trainee carpenter
Fitter	at least 4 years as a worker in shipbuilding, repair, or installation of hydraulic systems; or obtained the qualification of mechanical worker; or have completed 24 months of sea service as a trainee fitter or 36 months of sea service as a deck hand
Armament Captain	at least 3 years of sea service as a sailor
Boatswain/Second Boatswain	at least 4 years of sea service as a sailor
First Boatswain	at least 12 months of sea service as a boatswain
**ENGINE ROOM PERSONNEL**	
Engine Room Boy	18 years old
Ordinary Seaman (Engine Room)	at least 24 months of sea service, including 12 months in the engine room
Refrigeration Apprentice	a course at a professional training center for refrigeration technicians; or demonstrate with suitable documentation from a previous employer knowledge of refrigeration systems or air conditioning systems
Refrigeration Technician	a graduation from a State professional institute for maritime activities, specializing in refrigeration technicians; or have worked for at least 4 years as a refrigeration worker in production or repair facilities of refrigeration systems; or have completed at least 24 months of sea service as an Ordinary Seaman in cargo ships equipped with cargo refrigeration systems; or have completed at least 24 months of service on passenger ships assisting the refrigeration technician; or possess the qualification of motorist laborer; or have completed 24 months of sea service as a trainee refrigeration technician or 30 months of sea service as a refrigeration apprentice
Electrician Apprentice	a course at a professional training center for electricians; or have worked for at least 12 months in an electromechanical workshop or facility
Electrician or Second Electrician	a diploma from a State professional institute for maritime activities, specializing in electricians or electromechanics; or have served for at least 4 years in an electrotechnical establishment as an electrician; or have completed 24 months of sea service as an electrician apprentice or trainee electrician, or 24 months of sea service in the engine room with specific assignment to category services after attending a professional course
First Electrician	at least 12 months of sea service as an electrician or second electrician
Motorist Laborer	worked for 12 months in a naval mechanical workshop and 12 months of sea service operating internal combustion engines with a power not less than 50 HP/axis; or have completed, before or after attending a suitable professional course, at least 12 months of sea service as an Ordinary Seaman in the engine room; or have completed 24 months of sea service as a trainee motorist laborer
Mechanical Worker	a diploma from a State professional institute for maritime activities, specializing in mechanics, or hold the qualification of skilled or qualified worker with at least 12 months of service in facilities with such qualification; or have completed 24 months of sea service as a trainee mechanical worker
Chief Worker	24 months of sea service as a mechanical worker or come from the qualification of chief stoker
**MULTIPURPOSE PERSONNEL**	
Apprentice Multipurpose Seaman	18 years old and belong to the initial categories of Deck Boy, Deck Hand, or have maritime diplomas in deck and engine departments, and must possess a health booklet if assigned to kitchen or mess duties
Multipurpose Seaman	18 years old and have completed 24 total months of sea service in the initial qualifications, with at least 12 months on deck, and must have completed one of the courses for all-purpose seamen as required by Circular No. 88 of December 14, 1988, and its subsequent amendments and modifications; or have completed 24 months of sea service as a trainee all-purpose seaman
Trainee Multipurpose Worker	18 years old and have obtained a diploma from the State Professional Institute for Maritime Activities, machine section; or have a middle school license with at least 6 months of onboard work experience in the initial category
Multipurpose Worker	24 months of sea service as a trainee all-purpose worker or, after obtaining the qualification of mechanical worker, motorist laborer, refrigeration worker, or deck laborer, have completed 36 months of sea service (of which at least 12 months on deck to participate in navigation watch on the bridge) and must have completed one of the courses for all-purpose workers as required by Circular No. 88 of December 14, 1988, and its subsequent amendments and modifications

Chief Multipurpose Worker	at least 24 months of sea service as an all-purpose worker and must have completed one of the courses as required by Circular No. 88 of December 14, 1988, and its subsequent amendments and modifications
**HEALTH PERSONNEL**	
Nurse	recognition of the professional qualification
**SECOND CATEGORY**	
**ROOM PERSONNEL**	
Cabin Boy	16 years old
Second Steward	at least 12 months of sea service as a Cabin Boy
Steward	at least 18 months of sea service as a Cabin Boy, or at least 6 months as a Cabin Boy after attending a recognized hotel course
First Steward	at least 6 months of actual sea service as a Second Steward
Waiter	18 years old and have completed at least 12 months of actual sea service as a First Steward or Steward, or prove to have served at least one year in a hotel, or have accumulated at least 24 months of sea service as a trainee: commis, cabin steward, lounge steward, and ordinary room steward
Waiter (with good language skills)	18 years old and have completed at least 12 months of sea service as a First Steward with good language skills; or prove to have served in a hotel for at least 2 years with good language skills, or have accumulated 24 months of sea service as a trainee in hotel common, cabin steward, lounge steward, etc
Storeroom Waiter	at least 12 months of sea service as a Waiter
Head Steward - Accommodation Supervisor	at least 48 months of sea service as a Waiter
Assistant Wardrobe Keeper	18 months of sea service as a Cabin Boy
Wardrobe Keeper	at least 24 months of sea service as a Waiter; or 48 months of sea service as a First Steward or Steward
Butler - Maître d'hôtel	at least 24 months of sea service as a First Steward; or have served for at least 48 months as a butler in luxury or first-class hotels
Nanny	18 years old, have obtained a lower secondary school diploma
Bartender	at least 24 months of sea service as a Waiter, or at least 48 months of sea service as a Cabin Boy or Second Steward
Hotel Night Guard	24 months of sea service, including at least 6 months in the room section, and have successfully completed a recognized fire safety course
**COOKING AND HOUSEHOLD STAFF**	
Kitchen Assistant	must possess a health certificate
Kitchen Boy	at least 12 months of navigation as a kitchen assistant (this requirement is not necessary for cargo ships)
Third Cook	18 years old and have completed at least 24 months of navigation as a kitchen boy; or have completed 24 months of navigation as an apprentice cook
Second Cook	at least 24 months of navigation as a third cook or 36 months as a kitchen boy
First Cook/Station Chef	at least 24 months of navigation as a second cook
Sous Chef	at least 12 months of navigation as a first cook, or have completed at least 36 months of service as a cook in first-class hotels
Head Cook/Chef	at least 12 months of navigation as a sous chef or 24 months as a first cook, or demonstrate having served as head cook in large hotels
Crew Cook	the certification to embark as a ship's cook according to the decree of the President of the Republic on July 14, 1957, No. 1065
Crew Steward	at least 12 months of navigation as a crew cook
Pastry Assistant	must have the qualification of a kitchen boy or have completed 12 months of navigation as a kitchen assistant, or possess a certificate proving the exercise of the duties of a pastry assistant ashore
Pastry Chef	at least 12 months of navigation as a pastry assistant or possess a certificate proving the exercise of the duties of a pastry chef ashore, or have completed 24 months of navigation as an apprentice pastry chef
Head Pastry Chef	at least 12 months of navigation as a pastry chef or demonstrate having served as head pastry chef in large hotels
Pantry Boy	must possess a lower secondary school diploma and have completed at least 12 months of navigation as a kitchen boy, or 24 months of navigation as a kitchen assistant
Pantry Steward	at least 24 months of navigation as a pantry boy, or possess the qualification of a second cook, or have completed 24 months of navigation as an apprentice pantry steward
First Pantry Steward or Sole Pantry Steward	at least 24 months of navigation as a pantry steward, or possess the qualification of a first cook or steward or have completed at least 48 months as a pantry boy
Wine Steward	at least 24 months of navigation as a pantry boy or 18 months of navigation as a waiter in the dining room
Chief Steward	at least 24 months of navigation as a steward or pantry steward, or have completed at least 5 years of navigation as a cook or first waiter
Baker's Assistant	must have the qualification of a kitchen boy or have completed 12 months of navigation as a kitchen assistant, or possess a certificate proving the exercise of the duties of a baker's assistant ashore
Baker	18 years old and possess a certificate proving the exercise of the duties of a baker ashore or have completed at least 24 months of navigation as an apprentice baker or have the qualification of a crew cook
Head Baker	at least 24 months of navigation as a baker
Butcher's Assistant	must possess the qualification of a kitchen boy or a certificate attesting 12 months of work experience in a butcher shop as a butcher's assistant
Butcher	at least 24 months of navigation as a butcher's assistant, or possess a certificate attesting specific professional ability, or have completed at least 24 months of navigation as an apprentice butcher or have the qualification of a crew cook
Head Butcher	24 months of navigation as a butcher
Laundry Assistant	must possess a certificate of professional ability
Launderer/Ironer	18 years old and possess a certificate of professional ability issued by a previous employer
Head Launderer	at least 24 months of navigation as a launderer
**SERVICE STAFF***	

Printer	18 years old, possess a professional qualification certificate, and have worked for at least 12 months at a printing shop, or have completed at least 24 months of navigation as an apprentice printer
Projectionist	18 years old and possess a professional qualification certificate
Office Assistant	must have a lower secondary school diploma and have worked for at least two years in an office specializing in the tourism sector and have a good knowledge of at least one foreign language; or have obtained a business secretary's license, a secretary of administration qualification, or a high school diploma, or have completed 24 months of navigation as an apprentice office assistant
Hostess	must possess a professional qualification certificate issued by the Italian Tourist Culture Center, or have obtained a diploma from a language high school or commercial institute for business experts and foreign language correspondents; or provide appropriate documentation demonstrating at least 24 months of experience in tourist assistance at a sector agency or have navigated for 24 months as an apprentice hostess
**APPRENTICE NON-COMMISSIONED OFFICERS****	
**Deck section**	
Apprentice Tankerman	12 months of navigation in deck or engine room service
Apprentice Plumber	must possess suitable documentation proving at least 12 months of work experience as a plumber with a company, or have successfully completed a specific professional course
Apprentice Carpenter	must possess suitable documentation proving at least 12 months of specific work experience in a shipyard for the construction, repair, or outfitting of ships or floating vessels, or have successfully completed a specific professional course
**Engine Room Section**	
Apprentice Refrigeration Technician	a course at a professional training center for refrigeration technicians, or provide suitable documentation from a previous employer proving knowledge of refrigeration or air conditioning systems
Apprentice Motor Mechanic	a course at a professional training center for motor mechanics, or have worked for at least 12 months at a mechanical workshop or establishment
Apprentice Mechanical Worker	a course at a professional training center for mechanics, or have worked for at least 12 months at a mechanical workshop or establishment
Apprentice Electrician	a course at a professional training center for electricians, or have worked for at least 12 months at an electromechanical workshop or establishment
**Kitchen Section**	
Apprentice Provisioner	a lower secondary school diploma
**VARIOUS SERVICES SECTION****	
Apprentice Office Assistant	a lower secondary school diploma and have a fair knowledge of a foreign language
Apprentice Printer	18 years old and have worked for at least 12 months at a printing shop, or have successfully completed a specific professional course
Apprentice Hostess	a high school diploma
**HOTEL SERVICE APPRENTICES****	
**Room Section**	
Apprentice Room Steward	18 years old and possess a hotel school diploma, or a certificate of attendance from a professional hotel institute, or have a lower secondary school diploma with at least six months of onboard work experience in the initial category of cabin boy or room attendant
Apprentice Cabin Steward	a hotel school diploma or a certificate of attendance from a professional hotel institute
Apprentice Lounge Steward	a hotel school diploma or a certificate of attendance from a professional hotel institute
**Kitchen Section**	
Apprentice Cook	18 years old and possess a hotel school diploma or have a lower secondary school diploma with at least six months of onboard work experience in the initial category of kitchen boy or kitchen attendant
Apprentice Pastry Chef	a hotel school diploma or a certificate of attendance from a professional hotel institute
Apprentice Baker	a hotel school diploma or a certificate of attendance from a professional hotel institute
**THIRD CATEGORY**	
Personnel assigned to local traffic and coastal fishing	

* Note: For certain professional roles related to personal aesthetics, social entertainment, recreational activities, and passenger shopping such as beautician, manicurist, hairdresser, barber, gymnast, musician, social entertainer, salesperson, etc., boarding is permitted through the employment office at the shipowner's request, who evaluates the professionalism.

IMO STCW/78 Convention Compliance: In accordance with the IMO STCW/78 convention, adopted into national legislation by Law 21 November 1985, No. 739, deck and engine room personnel, as well as polyvalent personnel, must possess the certification required by the convention

** For the aforementioned apprentice qualifications, training and work contracts can be applied in accordance with Law No. 863/1984 and its subsequent amendments.
