# Supplementary material for: The Medico-Legal and Social Aspects of the Eligibility Examination for Enrolment in the Seafarers Registry: A Single-Center Retrospective Study
Source: Healthcare (Basel). 2024 Nov 30;12(23):2410. doi: 10.3390/healthcare12232410 (PMC11641269; doi:10.3390/healthcare12232410)
Supplement: Supplementary file 1 [file healthcare-12-02410-s001.zip › Supplementary Material Table S2.pdf]

Supplementary materials:

Table S2. **Supplemental materials:** List of illnesses and physical impairments that disqualify individuals from enrolment in the first category of the seafarers' register.

Art. 1	Constitutional weakness, developmental delays, and deformities that significantly impair the ability to endure the physical demands of onboard duties and exposure to disease-causing factors
Art. 2	A chest circumference less than 78 cm. For individuals with a tall, slender build (height of 1.75 m), the chest circumference must be no less than 84 cm
Art. 3	Obesity to a degree that significantly reduces agility and physical performance, especially when associated with short stature, accompanied by respiratory or circulatory disorders, and a significant predominance of abdominal circumference over chest circumference
Art. 4	Diabetes mellitus and other manifest disturbances of metabolic processes
Art. 5	Pulmonary and extrapulmonary tuberculosis of any form, localization, or stage. Suspected tuberculosis cases after positive confirmation by dispensaries affiliated with provincial anti-tuberculosis consortia or diagnostic centres of the National Fascist Social Security Institute*
Art. 6	Severe hematological disorders
Art. 7	Severe manifestations of chronic exogenous intoxication
Art. 8	Chronic ulcers, fistulas, sinus tracts, multiple old scars even if well-healed, when their extent, location, or adhesions interfere with movement freedom or the function of important organs, thereby reducing work capacity, or when they constitute disfiguring deformities
Art. 9	Organic diseases of the brain and spinal cord, peripheral paralysis, and progressive amyotrophies that impair the function of major muscle groups and thereby reduce work capacity
Art. 10	Extensive, disfiguring skin diseases, whether parasitic or not. Venereal and syphilitic diseases in the contagious phase
Art. 11	Malignant tumours and benign tumours that, due to size, number, or location, cause noticeable deformity or impede movement freedom and the function of an important organ, thus markedly reducing work performance
Art. 12	Muscle hernias, muscle and tendon ruptures, contractions, retractions, and muscular, tendinous, or aponeurotic adhesions that significantly impair movement freedom and markedly reduce work capacity
Art. 13	Chronic alterations of bones, joints, and periarticular tissues that manifestly disturb the function of an important organ or hinder the free movement of a limb or constitute a significant congenital or acquired deformity
Art. 14	Mutilations, inequalities, deviations, or deformities of a limb or limb segment that prevent the free and complete use of the limb and reduce the individual's work efficiency or constitute a noticeable deformity
Art. 15	Aneurysms of any type and grade, varicose veins that, due to their extent, volume, or location, cause noticeable and significant disturbances in organ function or the free and prolonged use of a limb
Art. 16	Obvious cretinism and idiocy, significant mental weakness, and psychological deficiencies that reasonably suggest the individual is not always fully aware of their actions. Severe character and behavioural anomalies. Stuttering and other significant speech disorders
Art. 17	Confirmed mental illnesses, and those previously treated when the individual has been under care in a psychiatric hospital or a special health facility
Art. 18	Epilepsy in its various forms, neurasthenia, hysteria, sleepwalking, and other neuroses when they present significant symptoms
Art. 19	Endocrine dysfunctions that cause pronounced circulatory or neurological disturbances or significant metabolic alterations
Art. 20	Acute or chronic conjunctivitis, particularly trachoma
Art. 21	Absence or obvious atrophy of an eyeball
Art. 22	Eye diseases and alterations that reduce visual function to the extent that visual acuity in both eyes is less than two-thirds.<br>[Visual acuity will be tested first in diffuse daylight and then in a dark environment, illuminating only the optotype characters of De Wecker**. Colour vision will be tested with coloured wool under diffuse daylight and then in a dark environment using coloured lanterns]<br><br>Whit the art. 1 of the Presidential Decree of April 30, 2010, No. 114 was amended the Art. 22 of the List and replaced by the following: "22. Diseases and alterations of the eye for which visual function is reduced to such a degree as to have in both eyes:<br>a) Deck personnel: natural vision below a total of 14/10 with less than 5/10 for the weaker eye. Corrected vision must be 10/10 in each eye with the use of well-tolerated lenses.<br>b) Other onboard personnel: natural vision below a total of 8/10 with less than 3/10 for the weaker eye.<br>Severe color vision deficiencies: For deck personnel and electricians, color function must be evaluated with the Ishihara Plates.<br>Malformations, dysfunctions, diseases, or the consequences of injuries of the eyelids or eyelashes, even if limited to one eye, when they cause significant functional disturbances.<br>Malformations, chronic diseases, and the consequences of injuries of the glands and tear ducts when they cause significant functional disturbances.<br>Defects in the visual field, even monocular, that significantly reduce vision. A visual field may be considered normal if it presents:<br>a) A total width on the horizontal meridian not less than 120°, on binocular evaluation.<br>b) A width on the vertical meridian of at least 60°, on binocular evaluation. Night blindness.
Art. 23	Chronic purulent otitis
Art. 24	Bilateral hearing loss when the voice is not perceived at a distance of less than 5 meters with a whisper (high-frequency phonemes), and unilateral hearing loss when the voice is not perceived at a distance of less than 1 meter
Art. 25	Cleft palate, ozena, significant chronic hypertrophy of the tonsils, and all permanent alterations of the mucosa and bones of the nose, adjacent sinuses, and mouth that disturb breathing.<br>N.B. – For radio operators, complete integrity of the pharyngeal, nasal, and upper airway structures must be required, including even modest signs of adenoidism
Art. 26	Goiter when it constitutes a significant deformity
Art. 27	The absence and extensive, deep decay of such a number of teeth that it severely disrupts pronunciation and the chewing mechanism, accompanied by dyspeptic disturbances affecting the overall health state
Art. 28	Recurrent asthma and all incurable respiratory conditions that constitute a permanent and severe alteration, functional or organic
Art. 29	Organic heart defects and cardiac neuroses
Art. 30	Severe or chronic abdominal organ diseases that cause functional alterations and affect the overall health state
Art. 31	All visceral hernias
Art. 32	Chronic urinary tract diseases that produce significant functional disturbances or alter the overall health state
Art. 33	All illnesses, defects, and functional disorders not specifically listed here that reduce work capacity, or the presence of various illnesses or imperfections each of which does not individually reach the severity described in previous articles, when collectively they manifestly impede the regular activities of the individual or are incompatible with the special requirements of onboard life***

* The National Institute for Social Security (INPS) was established in 1898 under the name National Fund for the Disability and Old Age of Workers. In 1933, the Fund became the National Fascist Institute of Social Security, a public entity with legal personality and management autonomy until the 1944, when it adopted its current name. ** The De Wecker optotype consists of a series of letters or symbols arranged in a way that progressively decreases in size, allowing for the measurement of visual acuity at various levels. *** The Royal Law Decree 1773/33 provides that Deck Personnel in the second category, responsible for watchkeeping, fuel storage, and fuel handling, must strictly adhere to all specified articles of the list. For Cabin and Service Personnel, especially waiters, both male and female staff serving passengers must meet certain physical appearance standards, “avoiding any traits that might repel guests”. For seafarers such as cooks, stewards, bakers, storekeepers, nurses, butchers, and messmen, the listed provisions should be applied with appropriate flexibility, considering their different working conditions.
