# Supplementary material for: The Medico-Legal and Social Aspects of the Eligibility Examination for Enrolment in the Seafarers Registry: A Single-Center Retrospective Study
Source: Healthcare (Basel). 2024 Nov 30;12(23):2410. doi: 10.3390/healthcare12232410 (PMC11641269; doi:10.3390/healthcare12232410)
Supplement: Supplementary file 1 [file healthcare-12-02410-s001.zip › Supplementary Material Table S3.pdf]

Table S3. **Supplemental materials:** STCW Code Table A-I/9 regarding the minimum in-service eyesight standards for seafarers.

STCW Convention regulation	Category of seafarer	Distance vision Aided[1]		Near/immediate vision	Colour vision[3,*]	Visual fields[4]	Night blindness[4]	Diplopia (double vision)[4]
		One eye	Other eye	Both eyes together, aided or unaided				
I/11 II/1 II/2 II/3 II/4 II/5 VII/2	Masters, deck officers and ratings required to undertake look-out duties	0,5[2]	0,5	Vision required for ship's navigation (e.g., chart and nautical publication reference, use of bridge instrumentation and equipment, and identification of aids to navigation)	See Note 6	Normal visual fields	Vision required to perform all necessary functions in darkness without compromise	No significant condition evident
I/11 III/1 III/2 III/3 III/4 III/5 III/6 III/7 VII/2	All engineer officers, electrotechnical officers, electrotechnical ratings and ratings or others forming part of an engineroom watch	0,4[5]	0,4 (see Note 5)	Vision required to read instruments in close proximity, to operate equipment, and to identify systems/ components as necessary	See Note 7	Sufficient visual fields	Vision required to perform all necessary functions in darkness without compromise	No significant condition evident
I/11 IV/2	GMDSS Radio operators	0,4	0,4	Vision required to read instruments in close proximity, to operate equipment, and to identify systems/ components as necessary	See Note 7	Sufficient visual fields	Vision required to perform all necessary functions in darkness without compromise	No significant condition evident

Notes:

1. Values given in Snellen decimal notation.
2. A value of at least 0.7 in one eye is recommended to reduce the risk of undetected underlying eye disease.
3. As defined in the International Recommendations for Colour Vision Requirements for Transport by the Commission Internationale de l'éclairage (CIE-143-2001 including any subsequent versions).
4. Subject to assessment by a clinical vision specialist where indicated by initial examination findings.
5. Engine department personnel shall have a combined eyesight vision of at least 0.4.
6. CIE colour vision standard 1 or 2.
7. CIE colour vision standard 1, 2 or 3.

* Regarding colour vision, the Code states that it must comply with the standards outlined in the International Recommendations for Colour Vision Requirements in Transport CIE 143:2001 (as reported in Table 2 of supplementary materials). This standard classifies individuals into four groups based on their colour vision capabilities: CIE 1 - Normal colour vision; CIE 2 - Slightly impaired colour vision; CIE 3 - Significantly impaired colour vision; CIE 4 - Non-classifiable, indicating more severe impairments that do not fit into the previous categories.
